# Esophageal Leiomyoma in Patients with Megaloblastic Anemia

**DOI:** 10.5005/jp-journals-10018-1110

**Published:** 2014-07-28

**Authors:** Adil Coskun, Mustafa Unubol, Ozden Yukselen, Vahit Yukselen, Ahmet Aydin, Serdar Şen, Ali Onder Karaoglu

**Affiliations:** 1 Department of Gastroenterology, Adnan Menderes University, School of Medicine, Aydin, Turkey; 2 Department of Endocrinology, Adnan Menderes University, School of Medicine, Aydin, Turkey; 3 Department of Pathology, Adnan Menderes University, School of Medicine, Aydin, Turkey; 4 Department of Gastroenterology, Ege University, School of Medicine, Izmir, Turkey; 5 Department of Thoracic Surgery, Adnan Menderes University, School of Medicine, Aydin, Turkey

**Keywords:** Esophageal leiomyoma, Megaloblastic anemia, Endoscopic assessment.

## Abstract

Esophageal leiomyoma is the most common benign intramural tumor of esophagus. Although its incidence is not exactly known, it is very rare (0.006%-0.1% in autopsy series). It is generally asymptomatic and detected incidentally. Here, we present a rare case report describing coexistence of megaloblastic anemia and esophageal leiomyoma.

**How to cite this article:** Coskun A, Unubol M, Yukselen O, Yukselen V, Aydin A, Şen S, Karaoglu AO. Esophageal Leiomyoma in Patients with Megaloblastic Anemia. Euroasian J Hepato-Gastroenterol 2014;4(2):98-100.

## INTRODUCTION

Leiomyoma is the most prevalent benign tumor of esophagus. It is very rare, and the incidence in autopsy series is 0.006 to 0.1%. It is mostly observed in the mid 1/3 or lower parts of esophagus. Its characteristic feature is the proliferation of the smooth muscle layer, causing circumferential thickening localized on the esophagus wall. Frequently, it is observed as a single lesion. It can be hereditary or sporadic. Half of the patients are asymptomatic. Symptoms are generally associated with dysphagia, nonspecific retrosternal pain, heartburn and rarely weight loss. Esophagography, chest X-ray, computerized thorax tomography, thorax magnetic resonance imaging (MRI), esophagogastroduodenoscopy are useful for diagnosis. Recently, transesophageal ultrasonography has become widely preferred for preoperative diagnosis.^1-3^ When esophageal leiomyoma is identified, lesion has to be removed even if the patient is asymptomatic. If the treatment is delayed or failed, the symptoms would probably develop and it will be hard to differentiate it from malignancy.^4^ Here, we present the case of a coexistence of megaloblastic anemia and esophageal leiomyoma.

**Fig. 1:**
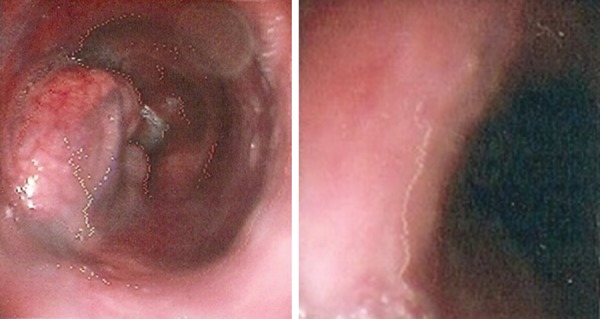
Esophagogastroduodenoscopy showing as a mass at distal esophagus, starting from incisor teeth, in between 34 and 37 cm, toward front wall

A 52-year-old male patient with previous history of asthenia and paresthesia before 4 years presented with low levels of blood hemoglobin (4.4 gm/dl). He showed a HCT value of 19.1%, MCV of 111.7 fl, RDW of 26.1%, leukocyte count of 2,900/ml, neutrophil count of 55%, lymphocyte count of 37%, monocyte count of 5%, eosinophil count of 3%, thrombocyte count of 123.000/ul In the peripheral blood smear of the patient who was diagnosed as pancytopenia, there were macrocytosis, anisocytosis in erythrocytes and hypersegmentation in neutrophils. Reticulocyte count was 1%. Serum B_12 _vitamin level was 88 pg/ml (normal range: 197-866 pg/ ml), being highly low. In the bone marrow aspiration and biopsy examination, cellularity of the bone marrow was 80% and the ratio between myeloid series and erythroid series was 1:1. Megaloblastic changes were observed in erythroid series. B_12_ treatment was started for the patient who was diagnosed to have megaloblastic anemia due to vitamin B_12_ deficiency. With a view of megaloblastic anemia, esophagogastroduodenoscopy was done. In the lower 1/3rd of esophagus between 34th and 37th cm, a protrusion was found into the lumen and which was covered with normal mucosa, and partially with sub-mucosal vascularization. It was lobulated in part and probably intramural (Fig. 1).

**Fig. 2:**
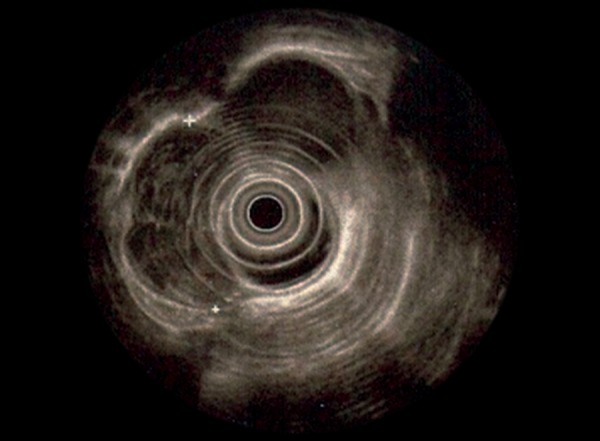
Endosonography showing a mass at distal esophagus 37.5 × 19.5 mm size, submucosally located, having smooth contours, heterogeneous hypoechoic and interpreted as compatible with mesenchymal tumor

The patient was planned to have endosonography, but did not show up for the follow-up. The patient came back after 4 years. The blood parameters showed hemoglobin: 13.8 gm/dl, HCT: 38.4%, MCV: 84,2 fl, leukocyte 5400**/m**l and thrombocyte 266.000**/m**l. The lesion was observed to have the same properties as before. The patient reported that he had developed no complaints in the meantime. Endosonography was performed. It was found as a mass at distal esophagus, starting from incisor teeth, in between 34th and 37th cm (37.5 × 19.5 mm in size), submucosally located, having smooth contours, heterogeneous hypoechoic and interpreted as compatible with mesenchymal tumor (Fig. 2).

**Figs 3A to C:**
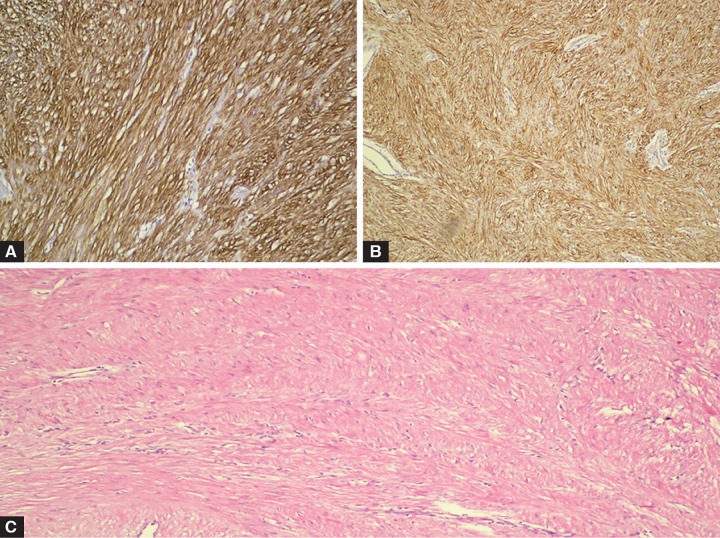
Staining pattern: (A) Actin (200x), (B) desmin (200x) and (C) hematoxylin and eosin (H&E: 100x)

The patient was consulted with thoracic surgeon for tissue diagnosis and tumor excision was planned. In the magnetic resonance examination of lung and mediastinum, after IV gadolinium (Gd), contrast involvement and circular wall thickening was observed in a segment of 4 cm at the distal end of esophagus. No extension toward surrounding soft tissues was found. In the biopsy sample, neoplastic cells were detected with actin and desmine, while negative with vimentin. Mitosis was not determined at sections, pathology result was determined as leiomyoma (Figs 3A to C).

## DISCUSSION

Less than 10% of esophagus tumors are benign tumors and 4% of them are comprised of leiomyoma.^3^ Esophageal leiomyoma constitutes 10% of all gastrointestinal system leiomyomas.^1^ Characteristic feature of leiomyoma is the proliferation of smooth muscle layer that causes circumferential thickening localized at esophagus wall. It is well-circumscribed, surrounded by a capsule of fibrous tissue, sessile, benign and spiral-shaped/whorled tumor. Tumor lesions are generally small, solitary with excentric position, solid, round and easily removable from the capsule. Generally, it is diagnosed between 20 and 50 years of age. It is observed more than twice in men than women.^5^ Our patient was a 52-year-old man. In 3 to 10% of cases, multiple numbers of lesions occur.^5^ Leiomyomas are usually intramural. However sometimes, these can be detected in the proximity of or inside of esophageal diverticulum.^6^ They can undergo cystic degeneration; however, progression to malignancy is rare. About 800 cases have been identified in the literature. Only in two of them, malignant transformation into leiomyosarcoma was observed (0.2%).^7^ In our case, the tumor lesion showed no change within 4 years. Nevertheless, leiomyomas accompanying/coexisting with malignant neoplasms have been defined in literature. It can be hereditary or sporadic. It has been related with Alport syndrome among hereditary diseases.^5^ If tumor exceeds mucosa and ulcer develops, bleeding may occur. In symptomatic patients, tumor diameter is observed (average 5.3 cm). In asymptomatic patients, its average diameter is about 1.5 cm.^1^ Generally, it grows slowly. The use of endoscopic ultrasonography in the diagnosis of esophageal leiomyoma is increasing.^3^ It is a considerably reliable method in differentiation of solid and cystic submucosal esophageal masses as well as in viewing these lesions. This method can be used in localizing mediastinal lesions and mural lesions of esophagus. In treatment, even if the leiomyoma is asymptomatic, it is advised to operate. Otherwise, malignancy cannot be eliminated. In differentiating from malignity, definite diagnosis must be done by excision and histological examination. Enucleation is a reliable and effective method of treatment. It provides relief in all symptomatic patients. Perioperative morbidity and mortality is not observed.^1^ In its monitoring, malignant transformation and recurrence tendency has not been observed. Intra-operative esophagoscopy combined with video-assisted thoracoscopic approach is the method used for easing the process and shortening the length of hospitalization. However, removal of all small asymptomatic leiomyoma may be unnecessary. In megaloblastic anemia, various idiopathic cases, such as gastric cancer and carcinoid, can be observed in gastrointestinal system.^8,9^ A single endoscopy should be considered to identify prevalent lesions (gastric cancer, carcinoid tumors) in patients with pernicious anemia, but there are insufficient data to support routine subsequent endoscopic surveillance for these patients.^10^ In case of megaloblastic anemia if gastrointestinal symptoms are seen, upper endoscopic examination may be useful.
